# Simultaneous resection of a gastric submucosal lipoma in the setting of bariatric surgery: A case report and review of current literature

**DOI:** 10.1016/j.ijscr.2019.06.031

**Published:** 2019-06-20

**Authors:** Slava Agafonoff, Tracy Pitt, Joshua Max, Steven Udelhofen, Timothy S. Braverman, Robert S. Lenobel

**Affiliations:** a The Jewish Hospital, 4777 E. Galbraith, Cincinnati, OH, United States; b The Jewish Hospital, United States; c TriHealth Digestive Institute, United States; d The Jewish Hospital – Pathology Department, United States; e The Jewish Hospital – Radiology Department, United States

**Keywords:** Sleeve gastrectomy, Endoscopic ultrasound, Submucosal lipoma, Endoscopic mucosal resection

## Abstract

**Introduction:**

Gastric submucosal lipoma is an uncommon finding in the stomach. A benign fatty tumor that is asymptomatic majority of the time. However, symptomatic and bigger tumors can be difficult to manage surgically and require detailed surgical planning prior to proceeding with bariatric surgery.

**Presentation of case:**

58 year old female who presented for weight loss consultation. Underwent an esophagogastroduodenoscopy (EGD) that demonstrated a 3 cm mass near the incisura. Endoscopic ultrasound (EUS) and biopsy demonstrated a submucosal lipoma. Due to the lipoma's location, resection was critical, as it would have led to obstructive symptoms following sleeve gastrectomy. The patient underwent a simultaneous laparoscopic vertical gastrectomy, gastric lipoma excision, EGD, and laparoscopic cholecystectomy

**Discussion:**

Gastric lipomas are benign gastric submucosal tumors, representing less than 3% of all benign gastric neoplasms. Computer tomography and endoscopic ultrasound are important in establishing diagnosis. In current literature, excision with negative margins is standard of care, but small asymptomatic lesions can be followed without intervention.

**Conclusion:**

Gastric lipoma are a rare type of gastric submucosal tumors. Size is highly variable. Observation is a reasonable approach when small and asymptomatic, but multiple surgical modalities can be utilized to remove the tumor. Careful utilization of pre-operative imaging including EUS is critical in choosing the appropriate surgery if simultaneous bariatric management is undertaken

## Highlights

• Important to rule out other tumors, such as GIST and malignancy.
• Pre-operative imaging including EUS is critical in choosing the appropriate surgery.
• Multiple modalities exist for excision of lipoma in setting of bariatric surgery.

## 1 Introduction

Gastric Lipomas account for 3% of gastric submucosal tumors (GST). When GST are found on pre-surgical endoscopy, definitive diagnosis can be challenging and affects surgical planning, requiring consideration of tumor location, type and consequent optimization of surgical modality. Preoperative diagnosis is imperative, as exclusion of atypical/malignant gastrointestinal tumors including gastrointestinal stromal tumor (GIST) is required for appropriate surgical planning.

This case report was reported in accordance with the SCARE criteria [12]

## 2 Case presentation

A 58 year-old female with a past medical history of diabetes mellitus and thyroid disease presented for weight loss consultation. There was no other pertinent surgical history. Prior to her endoscopy, her only subjective finding was mild occasional gastrointestinal reflux disease (GERD).

The patient underwent a pre-operative esophago-gastro-duodenoscopy (EGD) and was found to have a 3 cm submucosal mass along the anterior aspect of the gastric body, near the incisura angularis; biopsy at that time was indeterminate. As such, the patient was referred to gastroenterology for repeat EGD and endoscopic ultrasound (EUS).

**Fig. 1:**
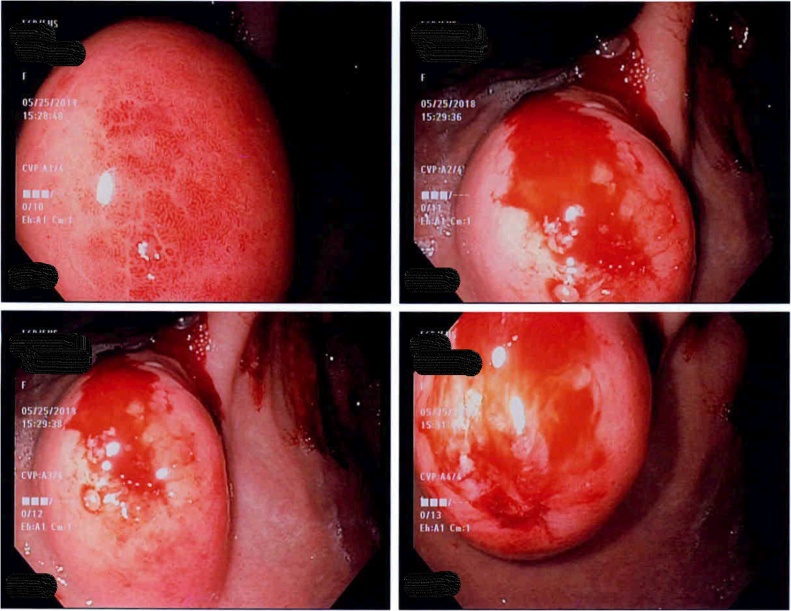
Esophagogastroduodenoscopy with a 3 cm mass along the anterior wall the gastric body at the level of the incisura. This had a positive pillow sign.

**Fig. 2:**
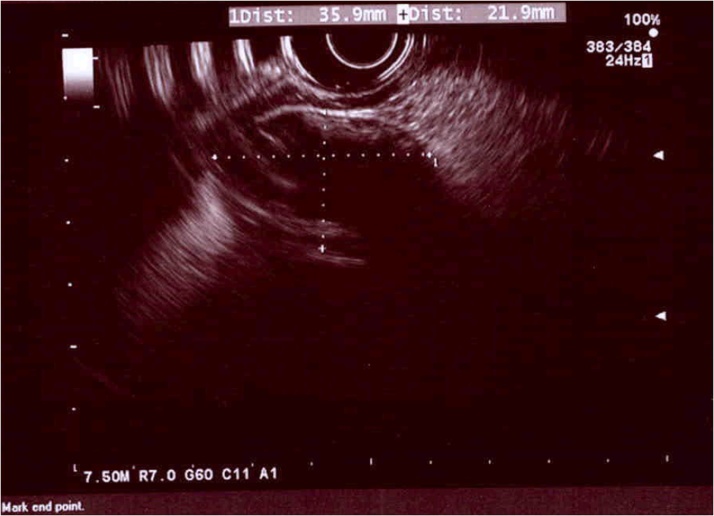
endoscopic ultrasound demonstrates a 3.3 x 1.6 cm relatively echogenic mass seeming to arise from the gastric submucosa in the area of the gastric lesion most consistent with benign lipoma.

**Fig. 3:**
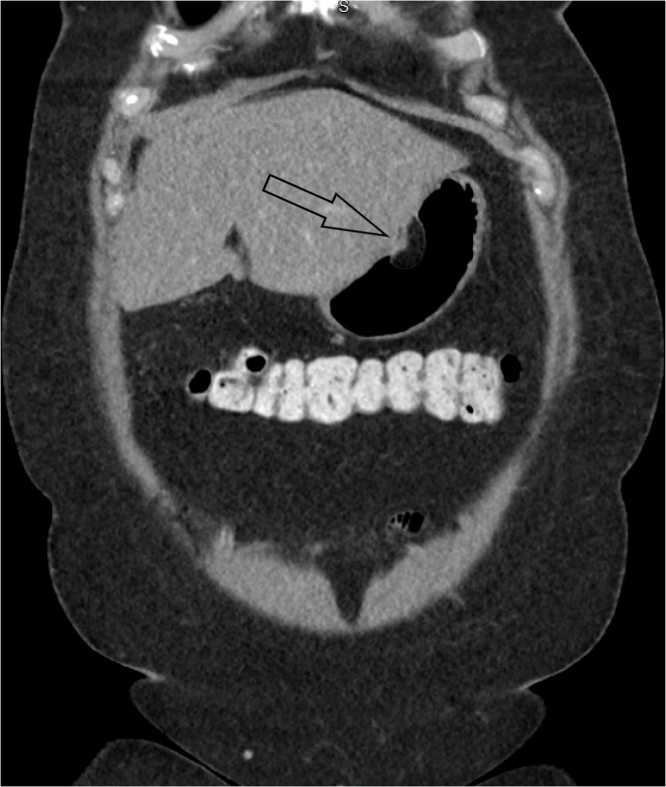
coronal post-contrast CT images show a well-circumscribed, smoothly marginated, ovoid intramural (submucosal) mass of uniform fat attenuation, arising from the lesser curvature of the body of the stomach with endoluminal extension. The mass measures 1.5 × 1.5 × 3.0.

Subsequent EGD re-demonstrated a 3 cm mass near the incisura. This had a positive pillow sign (pillowing of the surface of the lesion when prodded). Stack biopsies were obtained, with apparent fat within the biopsy, consistent with lipoma (Fig. 1). EUS was performed, revealing a 3.3 x 1.6 cm relatively echogenic mass arising from the gastric submucosa in the area of the gastric lesion consistent with lipoma (Fig. 2). Pathology was consistent with submucosal lipoma. Ancillary H. pylori biopsy was negative. CT scan re-demonstrated the lesion, confirming a lack of extra-gastric component (Fig. 3).

Due to the lipoma's location, resection was critical, as it would have led to obstructive symptoms following sleeve gastrectomy. The patient underwent a simultaneous laparoscopic vertical gastrectomy, gastric lipoma excision, EGD, and laparoscopic cholecystectomy. A longitudinal gastrotomy was made on the greater curvature. Exposure was achieved using retraction sutures. Following this, a laparoscopic submucosal resection of the lipoma was performed. We then used electrocautery to open the mucosa overlying the lipoma. Blunt and ultrasonic dissection were used to mobilize the lipoma. We then identified the vascular pedicle and divided this using the harmonic scalpel. A primary closure of the mucosa and greater curvature followed. The gastrocolic and gastrocolic omentum was taken down using the harmonic scalpel. We then used the linear cutting staple to perform the sleeve gastrectomy. Concurrent endoscopy was performed during the first two stapler firings to ensure that the mucosal suture line was not crossed by the stapler. We then inserted a 40 French Bougie and completed the sleeve in standard fashion. At 3 month follow up the patient was doing well.

## 3 Discussion

Gastric lipomas are benign gastric submucosal tumors, representing less than 3% of all benign gastric neoplasms. They are a proliferation of mature adipocytes occurring primarily in the submucosal layer, with 5–10% being subserosal. A majority are found in the fifth or sixth decade of life. The colon is the most common gastrointestinal location [1,2]. Most are asymptomatic, small, innocuous, and slow growing, however, depending on size (usually greater than 4 cm), gastric outlet obstruction, intussusception, abdominal mass, diarrhea, constipation, and abdominal pain may occur. Additionally, disabling dyspeptic symptoms have been reported. Hemorrhage is thought to occur secondary to the lipoma’s contact with the opposing wall, producing ulceration and necrosis [3,4].

The differential diagnosis includes a number of benign and malignant mural neoplasms, including GIST, carcinoid, neural tumor, and metastatic deposit. [3]

Lipomas can be difficult to differentiate from other submucosal lesions. Since the lesion occurs in the submucosal plane, endoscopic biopsy is unlikely to be diagnostic. Some endoscopic signs that can guide diagnosis include a ‘tenting sign’ which occurs when the normal mucosa overlying the lipoma is retracted easily away from the mass using biopsy forceps, pillow sign occurs when the forceps produces a soft, cushioning indentation when applied to the lipoma, and a ‘naked fat’ sign, which refers to exposed lipoma that protrudes through the normal overlying mucosa after multiple mucosal biopsies are performed [5]. Preoperative histological diagnosis can be more definitely established with endoscopic ultrasound (EUS)-guided fine needle aspiration for cytology. On EUS, gastric lipoma appear as a homogeneous, hyperechoic lesion arising from the third layer of the gastric wall [1]. In our case, diagnosis was confirmed by stack biopsies which provided adequate sample as well as classic appearance on EUS.

Computed tomography (CT) allows for a definitive diagnosis of gastric lipomas because of their uniform fat attenuation measuring from -80 to -120 Hounsfield units. Gastric lipomas on CT usually are a well-circumscribed, smoothly marginated, ovoid or spherical masses of homogenous low attenuation [2,[7], [8], [9]]. Gastric lipomas are intramural, with approximately 90–95% arising in the submucosa and the remaining 5–10% being subserosal. They grow towards the lumen and may be sessile or pedunculated [[9], [10], [11]]. Gastric lipomas are seen most commonly in the gastric antrum, although they may occur in the fundus and body of the stomach as well [1,3,5]. Occasionally linear strands of soft-tissue attenuation can be seen at the base of the lipoma on CT, which correspond histologically to fibro-vascular septa [10]. The soft-tissue attenuation at the base of the mass may be associated with overlying ulceration of the lipoma, which can cause gastrointestinal bleeding [10].

**Fig. 4:**
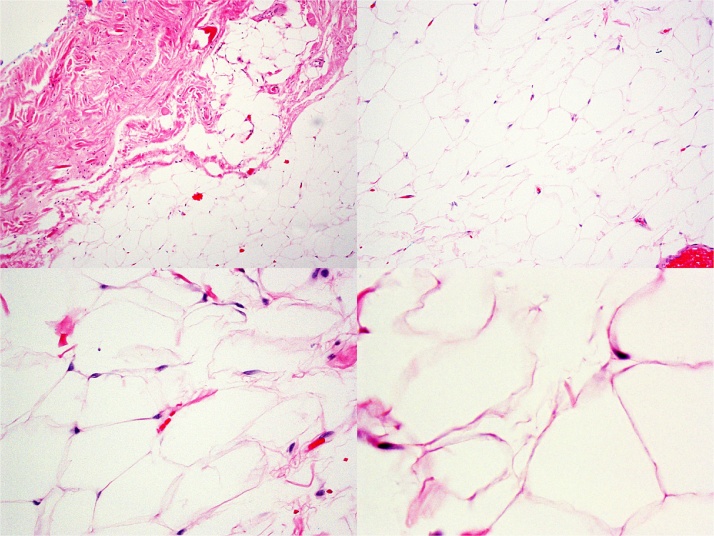
A typical encapsulated lipoma composed exclusively of mature adipocytes.

In pathology, lipoma, a benign fatty tumor, can arise in any location in which fat is normally present (Fig. 4). Most are subcutaneous, but they can also occur in deep soft tissues (including intramuscular), and may grow to large size, in some locations contributing to obstruction or other clinical symptomatology. They are commonly encapsulated when superficial, but often poorly circumscribed when deeper. Grossly and microscopically, they resemble normal fat. When accompanied by fat necrosis (infarction, with or without calcification), accompanying histiocytes must be differentiated from lipoblasts, which occur in lipoblastoma and hibernoma. Diffuse and multiple forms of lipoma are known, as are several variants with partial fibrous, myxoid, chondroid, muscular, spindle cell, vascular, and pleomorphic appearances. Cytogenetically, a majority of solitary benign lipomas show chromosomal abnormalities affecting primarily 6p, 12q, and 13q. Significantly lesser marker chromosomes similar to those typically seen in atypical lipomas, and MDM2 in well-differentiated liposarcoma, are seen, an important distinction between these tumors, which may closely morphologically resemble each other. Case reports of lipomas dedifferentiating into liposarcoma likely represent an unrecognized malignant component rather than true transformation, with due consideration to those rare cases of genetic progression or additional genetic ‘hits’.

Given the size and asymptomatic nature of the lipoma in our patient, we considered non-operative management, however, the location of the lipoma would have interfered with our staple line for the sleeve gastrectomy. Furthermore, given its location at the incisura angularis, there was a risk for gastric obstruction at this level following sleeve gastrectomy.

In current literature, excision with negative margins is standard of care, but small asymptomatic lesions can be followed without intervention. In contradistinction, when greater than 4 cm, some surgeons advise resection given a greater likelihood of associated complications. Asymptomatic cases can be alternatively managed without treatment as long as their diagnosis is confirmed. Though generally treated with partial gastrectomy a number of reports document the successful use of endoscopic submucosal dissection (ESD) for resection. Ichinose et al., demonstrated a successful endoscopic resection for a 50 year -old male [5]

Kang et al. [4] demonstrated feasibility in performing laparoscopic-endoscopic cooperative surgery (LECS) for gastric submucosal tumors. Retrospectively reviewed in 101 patients, they successfully removed gastric submucosal tumors, two of which were diagnosed as lipomas. A combination of EGD and laparoscopy is a safe method when treating these lesions, as, as in our case where the lipoma was removed in concert with a sleeve gastrectomy.

Our case was unique with only one other published case report that involves simultaneous bariatric surgery and resection of a gastric submucosal lipoma. In that case Hashimoto et al., performed a laparoscopic roux-Y gastric bypass along with a laparoscopic intragastric surgery in a 43 year-old morbidly obese female [6].

There are multiple surgical approaches to excise a lipoma, however, in the setting of simultaneous bariatric surgery, careful pre-operative imaging and planning are critical.

## 4 Conclusion

Gastric lipoma are a rare type of gastric submucosal tumors. Size is highly variable. Observation is a reasonable approach when small and asymptomatic, but multiple surgical modalities can be utilized to remove the tumor. Careful utilization of pre-operative imaging including EUS is critical in choosing the appropriate surgery if simultaneous bariatric management is undertaken.

## Conflicts of interest

There are no conflicts of interest.

## Ethical approval

Ethical approval is exempted as this is a case report.

## Consent

Written/Verbal informed consent was obtained from the patient for publication of this case report and accompanying images. A copy of the written consent is available for review by the Editor-in-Chief of this journal on request.

## Funding

No source of funding.

## Author contribution

Dr. Agafonoff did the bulk of the paper.

Dr. Braverman contributed to the pathology section of the paper.

Dr. Linobel contributed to the radiology section of the paper.

Dr. Pitt/Dr. Udelhofen/Dr. Max contributed to the writing of the paper.

## Registration of research studies

Not applicable.

## Guarantor

Guarantor is Dr. Slava Agafonoff.

## Provenance and peer review

Not commissioned, externally peer-reviewed.
